# Do Metabolomics and Taxonomic Barcode Markers Tell the Same Story about the Evolution of *Saccharomyces sensu stricto* Complex in Fermentative Environments?

**DOI:** 10.3390/microorganisms8081242

**Published:** 2020-08-15

**Authors:** Luca Roscini, Angela Conti, Debora Casagrande Pierantoni, Vincent Robert, Laura Corte, Gianluigi Cardinali

**Affiliations:** 1 Department of Pharmaceutical Sciences, University of Perugia, 06121 Perugia, Italy; luca.roscini@unipg.it (L.R.); angelaconti21@gmail.com (A.C.); deboracasagrandepierantoni@gmail.com (D.C.P.); gianluigi.cardinali@unipg.it (G.C.); 2 Westerdijk Fungal Biodiversity Institute, Uppsalalaan 8, 3584 CT Utrecht, The Netherlands; vrobert@bio-aware.com

**Keywords:** Saccharomyces, metabolomic fingerprint, barcode markers, yeast taxonomy, FTIR, LC-MS metabolomic

## Abstract

Yeast taxonomy was introduced based on the idea that physiological properties would help discriminate species, thus assuming a strong link between physiology and taxonomy. However, the instability of physiological characteristics within species configured them as not ideal markers for species delimitation, shading the importance of physiology and paving the way to the DNA-based taxonomy. The hypothesis of reconnecting taxonomy with specific traits from phylogenies has been successfully explored for Bacteria and Archaea, suggesting that a similar route can be traveled for yeasts. In this framework, thirteen single copy loci were used to investigate the predictability of complex Fourier Transform InfaRed spectroscopy (FTIR) and High-performance Liquid Chromatography–Mass Spectrometry (LC-MS) profiles of the four historical species of the *Saccharomyces sensu stricto* group, both on resting cells and under short-term ethanol stress. Our data show a significant connection between the taxonomy and physiology of these strains. Eight markers out of the thirteen tested displayed high correlation values with LC-MS profiles of cells in resting condition, confirming the low efficacy of FTIR in the identification of strains of closely related species. Conversely, most genetic markers displayed increasing trends of correlation with FTIR profiles as the ethanol concentration increased, according to their role in the cellular response to different type of stress.

## 1. Introduction

Yeast taxonomy debuted at the beginning of the 20th century with a monography based on morphology and the analysis of a few physiological traits that were eventually increased by the Dutch School to the over 70 traits suggested in the various editions of “The Yeasts—a taxonomic study” [1,2,3]. The idea at that time was simply that the assimilation and fermentation properties would help discriminated species, thus assuming a strong link between physiology and taxonomy. Some milestones papers show that these characteristics can be instable and vary within the species, thus suggesting that they were not ideal markers for species delimitation and that the species is not a boundary of unique traits combinations. These observations helped molecular taxonomy to take off, with DNA–DNA reassociation [4] and then with the sequencing of the LSU marker [2]. In the meantime, the concept of taxonomy moved from a phenetic description of taxa to their phylogenetic reconstruction [5]. The overwhelming amount of molecular data produced in the last quarter of a century has shaded the importance of physiology. This has led to assimilate a taxon to its DNA and, more specifically, to its marker DNA, to the extent that the OTU (Operational Taxonomic Unit) are transformed in the MOTU (Molecular OTU) [6]. This approach allowed a rapid and relatively stable description of new species and, above all, paved the way to the concept of barcoding [7] and hence to metagenomics. The vast use of DNA for species delimitation and identification soon became the only system available to uncover the vast extent of viable but non-culturable biodiversity [8,9,10]. Parallel to this evolution of the DNA-based taxonomy, the advent of proteomics and metabolomics gave new impetus to the study of the phenotype in broad sense [11,12,13,14]. Whereas the application of artificial intelligence [15,16,17] will surely improve the ability of metabolomics and metabolomic fingerprinting tools in the species identification, the primary role of these analytical systems is their ability to finely characterize the physiological status in various conditions, including different types of stress [18]. The ability of microbial cells to withstand stress is part of the phenotypical traits used in species description with good discriminating ability both at the specific and at the subspecific level [19]. Beyond this purely taxonomic application, the response of cells to the stress is of primary importance in ecology and in industrial applications. This consideration poses the question on the role of the stress response in discriminating taxa over and, more importantly, below the species level.

Most of the efforts outlined above proved very effective in improving the taxonomic efficacy and its resolution [20], although the question remains on what is the real meaning of this species concept, especially when it should be applied to applicative fields where it is not so important to define the species, or its name, but rather to infer some basic characteristics of the strains from the fact that they belong to a given species. As long as the taxonomy was basically phenotype-based, several species were separated for one or few physiological characteristics, as it was in the case of the *Saccharomyces sensu stricto* group up to the 1984 revision by Yarrow, which merged all the species of the group in the single species *S. cerevisiae*, and then split them into four species (*S. cerevisiae, S. bayanus, S. paradoxus* and *S. pastorianus*) on the basis of DNA–DNA reassociation studies [21], later reinforced with chromosomal electro karyotyping [22]. When *S. cerevisiae, S. chevalieri* and *S. italicus* where merged in *S. cerevisiae* for their rDNA homology [4], their specific physiological and even oenological traits that had led to the delimitation of different species were hidden under a common name. It is not the everlasting phenomenon of splitting and lumping typical of taxonomic rearrangements, but rather a was a choice for the trait stability, and therefore in a way for the taxonomic stability, sacrificing the specificity of groups that could not be any more considered “species”. The choice by itself is taxonomically correct but takes away the fact that people in the applicative field associated species epithets with physiological and industrially relevant features.

In the current situation, the idea of turning back to phenotypic identification is far from optimal because it would strongly block all the studies on the viable and not culturable microbial diversity. However, reconnecting taxonomy with relevant phenotypic features is an important aim from both an applicative and a general point of view, which can be obtained by exploring the predictability of important features of the studied strains, using not only the traditional rRNA based markers ITS and LSU, but also other single copy genes that have been suggested as new generation markers.

Predictability of specific traits from phylogenies has been explored for Bacteria and Archaea with quite encouraging results for both binary and quantitative traits [23], suggesting that a similar route can be endeavored in yeast biology as well, considering their paramount importance as a model organism and in various applicative fields from oenology to biotechnology.

The aim of this paper is to investigate on the correlation between thirteen taxonomic markers and the LC-MS and FTIR profiles of the representative type strains of the four historical species the *Saccharomyces sensu stricto* group [22], in order to explore the predictability that each of these markers has with the complex phenotypic profiles obtained by these omic tools.

## 2. Materials and Methods

### 2.1. Cultures and Growth Conditions

The yeast strains used in this work are: *S. bayanus* CBS 380, *S. paradoxus* CBS 432, *S. cerevisiae* CBS 1171 and *S. pastorianus* CBS 1538. Cultures were inoculated at an optical density at 600 nm (OD_600_) of 0.2 in 500-mL bottles with 50 mL of fresh Yeast Nitrogen Base (YNB) medium supplemented with 5% glucose (Difco Laboratories, Franklin Lakes, NJ, USA) and grown at 25 ± 0.5 °C under shaking at 150 ± 1 rpm. Cell growth was monitored by determining OD_600_ and stopped after 18 h. Each culture was prepared for an FTIR-based bioassay, as detailed in the following paragraph.

### 2.2. FTIR-Based Bioassay

An FTIR-based assay for stress response analysis was carried out according to the procedure proposed by Corte and colleagues [18]. Each suspension was centrifuged (5 min at 5300 ± 10× *g*), washed twice with distilled sterile water and re-suspended in High Performance Liquid Chromatography (HPLC) grade water to obtain an optical density of OD_600_ = 50. Each cell suspension was distributed in 15-mL polypropylene tubes, one for each tested concentration of the chemicals. In each tube, 5 mL of cell suspension and 5 mL of double-concentrate ethanol solution were pipetted to obtain the final concentrations of 8%, 12% and 16% (*v*/*v*) and a uniform cell density at OD_600_ = 25. Control (0% ethanol concentration) was obtained by re-suspending cells in distilled sterile water. All tests were carried out in triplicate. Polypropylene tubes were incubated for 1 h at 25 °C in a shaking incubator set at 50 ± 1 rpm. After the incubation, 1.5 mL of each cell suspension were centrifuged (5 min at 5300 ± 10× *g*), washed three times with distilled sterile water and resuspended in HPLC grade water to reach the final concentration of 2.5 × 10^8^ cells mL^−1^. For each condition, 105 µL volume was sampled for three independent FTIR readings (35 µL each, according to the technique suggested by Essendoubi and colleagues [24] while 100 µL were serially diluted for viability assessment. Viable cell count was carried out on YPDA (Yeast extract 1%, Peptone 1%, Dextrose 2%, Agar 1.8%) supplemented with chloramphenicol (0.5 g L^−1^) plates. Cell mortality (M) was calculated as M = (1 − Cv/Ct) × 100, where Cv is the number of viable cells in the tested sample and Ct the number of viable cells in the control suspension.

### 2.3. Spectra Pre-Processing

FTIR measurements were performed in transmission mode. All spectra were recorded in the range between 4000 and 400 cm^−1^. Spectral resolution was set at 4 cm^−1^, sampling 256 scans per sample to obtain high quality spectra (signal to noise ratio values greater than 4000 within the 2100–1900 cm^−1^ interval). The software OPUS v. 6.5 (BRUKER Optics GmbH, Ettlingen, Germany) was used to assess the quality test, subtract the interference of atmospheric CO_2_ and water vapor, correct baseline (rubberband method with 64 points) and apply vector normalization to the whole spectra.

### 2.4. Untargeted Metabolomics Profile Determination of Yeast Cells by LC-MS Analysis

The metabolomic analysis of CBS 380, CBS 432, CBS 1171 and CBS 1538 samples at increasing ethanol concentrations (0%, 8%, 12% and 16%, *v*/*v*) was performed using a LC-MS untargeted workflow. Each thesis was tested in five replicates (*n* = 5), with a total of 80 samples processed. Cell suspensions, calibrated at 10^8^ cells, was centrifuged (5 min at 5300 ± 10× *g*) and the resulting pellet was mixed with glass beads and lysed using FastPrep^®^-24 Tissue and Cell Homogenizer (MP Biomedicals, Irvine, CA, USA), at a speed setting of 6.0 for 120 s. The degree of cell breakage was checked microscopically. One milliliter of methanol was added to each lysate, vortexed and centrifuged at 3000 rpm for 5 min. Supernatants were transferred to the HPLC vials and 0.5 µL was injected into the LC-MS system. LC-MS analyses were performed using an Agilent 1260 Infinity UHPLC system coupled to an Agilent 6530 Q-TOF with Agilent JetStream source (Agilent Technologies, Santa Clara, CA, USA). The LC consists of a quaternary pump and an autosampler with a thermostated column compartment. The whole LC-MS system was governed by Agilent MassHunter software (v. B.09.00). The Ion Pairing Chromatography (IPC) method was used to achieve a wide separation of polar metabolite classes with ACME^TM^ Amide C18 column (150 × 2.1 mm, 3 µm, Phase Analytical Technology LLC, State College, PA, USA) thermostated at 50 °C. The separation of metabolites was achieved using a flow of 0.35 mL min^−1^ of a binary gradient of 0.2% heptafluorobutyric acid (HFBA) in water (Solvent A) and 0.1% formic acid in methanol (Solvent B). The initial condition was 2% of B for 2 min, followed by a transition to gradient from 2% to 80% of B in 5 min and an isocratic step of 8 min. After that, the run was stopped, and the column was reconditioned for 4 min at initial conditions. An autosampler injected each sample using a needle wash program of 10 sec with methanol. Each run cycle was completed in 20 min. The ion source operated in positive ion mode using nitrogen as drying gas at 35 psi and 250 °C. The capillary was set at 2000 V with fragmentor, skimmer and octopole Radio Frequency (RF) set at 110, 65 and 750, respectively. Dynamic mass axis calibration with accuracy < 5 ppm was achieved by continuous infusion in the source of a reference mass solution (Agilent G1969-85001). The spectrometer acquired data in full-scan mode in the 50–1700 mass range at 1.5 spectra/s. LC-MS raw files were aligned and processed using Batch Recursive Feature Extraction algorithm of MassHunter Profinder (Agilent B.08.00). The data of features with score > 90% were imported in Mass Profiler Software (Agilent B.08.01) to perform features annotation using the Search Database algorithm. For this purpose, the Yeast Metabolome Database [25] was adapted to work in Agilent Mass Profiler. Only annotated metabolites with a quality identification score > 90% were retained.

### 2.5. Data Analysis

#### 2.5.1. Phylogenetic Analysis

Alignment of the concatenated ITS and D1/D2 domain of the 26S rDNA (LSU) sequences of the four strains was carried out with ClustalW2 built-in tool in MEGA X [26]. Distances were inferred with the Maximum Composite Likelihood method and expressed as number of base substitutions per site. This procedure has been chosen because it assumes equal substitution patterns and rates among lineages and sites, conditions considered appropriate for ongoing separation phenomena. Both transitions and transversions were considered. The Neighbor-Joining method [27] was used to reconstruct the tree with 1000 bootstrap reiterations.

#### 2.5.2. Correlation between Genetic Markers and Metabolomic Data

Correlation analysis between genetic and metabolomic markers was performed using a series of thirteen different loci which included the traditional ITS and LSU rRNA-based markers and other single copy genes suggested as new generation markers (Table 1).

For each strain, marker sequences were retrieved from whole genome assemblies using an ad-hoc pipeline, set up using freeware bioinformatic tools, as detailed in the following lines.

For each marker, reference FASTA sequences from *S. cerevisiae* S288C reference genome (obtained from Genbank) were aligned to each genome assembly and the coordinates of regions with positive matchings were retrieved using NUCmer function of MUMmer software [28,29]. Markers sequences were then extracted as FASTA files from these positive matchings using SAMtools software [30].

Markers alignments were carried out with ClustalW2 built-in tool in MEGA X [26]. Distances were calculated in R environment (http://www.R-project.org) using *dist.dna* function from APE R-package (http://ape.mpl.ird.fr/).

Correlation analysis between genotypic and metabolomic distance matrices from cells in resting condition was carried out by using the function *cor.test* included in the Vegan package (https://CRAN.R-project.org/package=vegan). Data were then exported and analyzed in MS Excel^TM^.

Correlation analysis between genetic vs. FTIR and LC-MS data from cells under stress was carried out by using the *dis.maca* function from the R script DADI [31]. Briefly, distance matrices of strains from each genetic marker were correlated with distance matrices from the first descriptor of each spectral data matrix, applying the default parameters (Euclidean distances and Pearson correlation calculation). The procedure was repeated for each descriptor of all spectral matrices. Correlation matrices were then exported and filtered in MS Excel^TM^. Correlations with FTIR data were analyzed considering both the whole IR spectrum and each of the characteristic spectral region from W1 to W4 [32,33].

#### 2.5.3. HCA and Pathway Analyses of LC-MS Data

The sample weight and non-normalized peak areas are available in Table S1.

Metabolomic data analysis was performed with MetaboAnalyst 4.0 [34]. Data were filtered based on Interquartile Range, normalized to sample median and scaled by Pareto scaling.

HCA was carried out to highlight the variation trend of differential metabolites between strains challenged by increasing ethanol concentrations setting the Euclidean correlation method and Ward clustering algorithm. Metabolites were selected according to the VIP scores from Partial Least Square-Discriminant Analysis (PLS-DA) (Tables S2–S5). Since the average of squared VIP score is equal to 1, the “greater than one” rule was used as a criterion for variable selection.

Pathway analysis was performed to discover the most relative pathways involved in the response of these strains to short-term ethanol stress (Tables S6–S9). The global test algorithm and relative-betweenness centrality algorithm were specified for pathway enrichment analysis and pathway topology analysis, respectively.

## 3. Results

### 3.1. Phylogenetic Predictability: Correlation between DNA-Based and Phenotypic Markers

To assess the relation between metabolomics and DNA taxonomic markers, FTIR and LC-MS were employed for the former analyses and a set of thirteen loci of taxonomic use for the latter (Table 1, M&M section). The choice of these markers was guided by the following criteria: the ITS, LSU and the concatenated ITS_LSU sequences were included as benchmark since they were used over the last 22 years as identification tool [2,7]; *DAL2*, *FAS1* and *ICL1* single copy genes were selected as genes involved in crucial metabolic pathways [35,36,37,38]; and *ACT1*, *mtCOXII*, *mtSSU*, *RPB1*, *RPB2*, *SSU* and *TEF1-α* sequences were selected because they are currently proposed as new generation markers for yeast taxonomy [7,39,40,41,42].

Out of these 13 loci, the concatenated sequence ITS_LSU was chosen for the preliminary investigation on the phylogenetic predictability of metabolomics profiles, whereas the whole set of markers was used for a second step analysis described below. The phenotypic analysis suffers traditionally of repeatability problems and is obviously sensitive to the growth phase at which cells are analyzed. In this respect, late exponential phase was chosen, because it can be easily detected from the growth curve.

**Figure 1:**
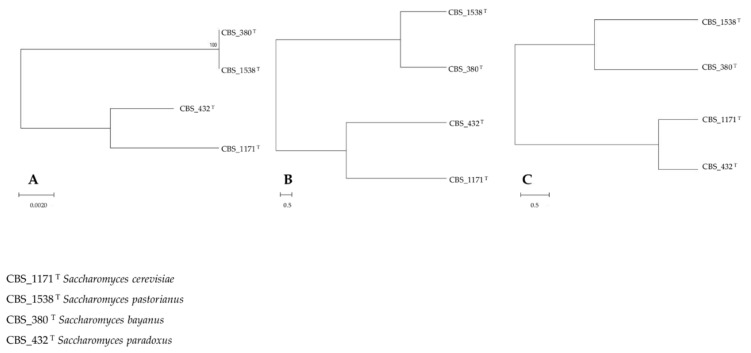
Genotypic vs. phenotypic FTIR and LC-MS description of *S. bayanus*, *S. pastorianus*, *S. paradoxus* and *S. cerevisiae* reference strains. Hierarchical clustering of *S. bayanus* CBS 380, *S. pastorianus* CBS 1538, *S. paradoxus* CBS 432 and *S. cerevisiae* CBS 1171 control samples (0% ethanol). (**A**) Clustering obtained analyzing concatenated ITS_LSU sequences; distances were inferred with the Maximum Composite Likelihood method and expressed as number of base substitutions per site. The Neighbor-Joining method was used to reconstruct the tree. (**B**) Clustering obtained analyzing FTIR spectra considering the regions from 3200 to 2800 cm^−1^ (fatty acids) and from 1800 to 1200 cm^−1^ (amides and mixed region). (**C**) Clustering obtained analyzing LC-MS spectra using Spearman’s distance measure and Ward’s algorithm. Hierarchical clustering of phenotypes was performed.

Interestingly, trees from cluster analysis of metabolomic data substantially reproduced the same clusterization of the strains obtained through their phylogenetic description with ITS_LSU barcode (Figure 1A). In fact, with both FTIR fingerprint (Figure 1B) and LC-MS metabolomics (Figure 1C) *S. bayanus* CBS 380 and *S. pastorianus* CBS 1538 clustered separately from *S. paradoxus* CBS 432 and *S. cerevisiae* CBS 1171. However, the distances among the strains provided by the ITS-LSU clustering were significantly different from those indicated by the phenotypic markers.

The substantial identity among *S. paradoxus* CBS 1538 and *S. bayanus* CBS 380, as for the ITS_LSU analysis, was not confirmed by means of FTIR and LC-MS, showing an increased distance among these strains, particularly marked in the LC-MS data cluster. Conversely, the distance between *S. paradoxus* CBS 432 and *S. cerevisiae* CBS 1171 was roughly the same when comparing ITS_LSU and FTIR clusters while it was reduced by the LC-MS description.

The discrepancies among the qualitative and quantitative representation of strains depicted in Figure 1 suggested to deepen the study by using the whole set of taxonomic markers (Table 1) to better evaluate if and to what extent the DNA-based markers can be effective in predicting the phenotypic profiles of these strains.

**Figure 2:**
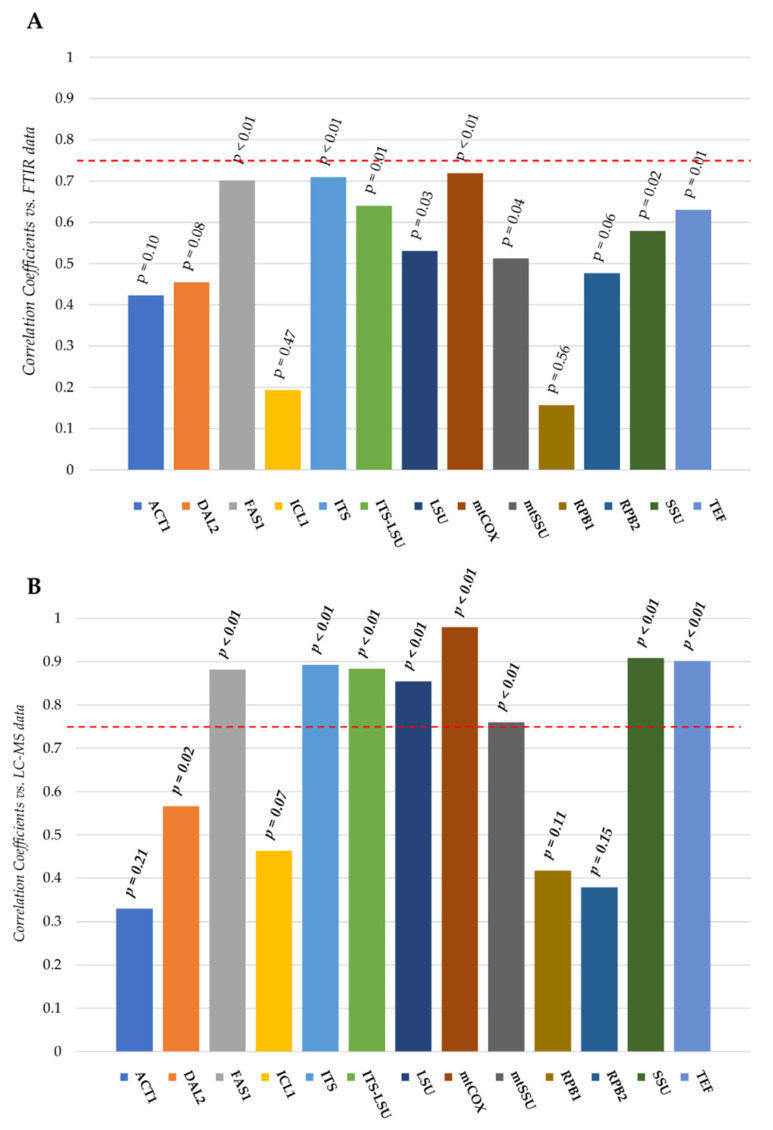
Correlation between taxonomic markers and phenotypic responses of control samples of *S. bayanus*, *S. pastorianus*, *S. paradoxus* and *S. cerevisiae* reference strains. Correlation coefficients obtained from the comparison between the distance matrices calculated on the basis of each of the thirteen loci employed in the study and those obtained analyzing FTIR (**A**) and LC-MS (**B**) spectra of strains in resting condition are reported. Distances were calculated using *dist* and *dist.dna* functions included in R-Ape package (https://cran.r-project.org/web/packages/ape/index.html). Correlation analysis was carried out using *cor.test* function included in the R-Vegan package (https://CRAN.R-project.org/package=vegan).

Distance matrices from FTIR and LC-MS spectra of each strain were correlated with those obtained for each locus included in the study (Figure 2).

Overall, the correlation between DNA-based and FTIR data (Figure 2A) was lower than that obtained for LC-MS (Figure 2B).

No significant correlations (correlation coefficients > 0.75) was detected among FTIR and taxonomic markers (Figure 2A). Conversely, eight markers out of the thirteen tested showed correlation coefficients higher than 0.75 with distance matrices from LC-MS spectra (Figure 2B). The mitochondrial subunit 2 of cytochrome oxidase (*mtCOXII*) gene was the marker that correlated best with the LC-MS data (0.97; *p* value < 0.01). High correlation values (≥0.9) were also registered for the 18S nuclear ribosomal small subunit (SSU) and the Translation Elongation Factor 1 alpha (*TEF1α*) sequences, with 0.91 and 0.90 coefficients, respectively (*p* values < 0.01). In addition, the *FAS1* (Beta subunit of fatty acid synthetase) and the small subunit mitochondrial rRNA (*mtSSU*) genes have proved to be good markers towards LC-MS profiles of the four strains (0.88 and 0.76 correlation coefficients, respectively; *p* values < 0.01). Interestingly, results obtained with traditional markers revealed that the ITS barcode showed a better correlation with these phenotypic data than the concatenated sequence ITS_LSU, which in turn displayed a better correlation than the LSU alone (0.89, 0.88, 0.85 correlation coefficients; *p* values < 0.01).

Finally, the analysis did not reveal any significant correlation between *RPB1*, *RPB2*, *ICL1* and *ACT1* coding sequences and these metabolomic phenotypic descriptors.

### 3.2. Stress Predictivity

The potential applicability of some of the mentioned above markers for the prediction of strains phenotypes in resting condition paved the way for another question on whether this set of taxonomic loci could be used to predict the phenotypes under stress. This hypothesis springs from the observation that these species evolved with a stringent selection due to the highly challenging environments with high alcohol content. At this aim, the genetic distances between the four strains, calculated for each of the thirteen markers employed in the study (Table 1), were compared with distances obtained from FTIR spectra of cells in response to short-term ethanol stress. More in detail, a FTIR-based assay, specifically designed for stress response assessment [18], was carried out on the whole cells of each culture at increasing ethanol concentrations (0%, 8%, 12% and 16% *v*/*v*), paralleling with viable analysis.

#### 3.2.1. Mortality Analysis

The analysis of cell mortality showed a strain-specific pattern in response to ethanol (Table 2). *S. bayanus* CBS 380 was the most sensitive strain with around 40% mortality already at the lowest ethanol concentration tested (8%), almost saturated at 12% ethanol. Conversely, ethanol did not significantly affect the viability of *S. pastorianus* CBS 1538 until 12%, which in any case remained over 90% at higher ethanol concentrations, suggesting a strong resistance for this strain. The mortality induced by ethanol on the other two strains, *S. paradoxus* CBS 432 and *S. cerevisiae* 1171, is close to that of *S. pastorianus* CBS 1538. In fact, the first displayed mortality values ranging from 11% to 13% for all the tested concentrations while the latter showed mortality values around 10% and 20% at 12% and 16% ethanol, respectively.

#### 3.2.2. Correlation between Molecular Markers and FTIR Metabolomic Fingerprints of Cells under Short-Term Ethanol Stress

The comparison between genetic data and FTIR profiles of ethanol stressed cells was carried out calculating Pearson’s correlations between the distances obtained considering each of the thirteen genetic markers and those obtained comparing each of wavelength of each spectral matrix. The resulting correlation matrices were filtered, in order to retain only the wavelength with a correlation higher than 0.75 (Table S10), and then expressed as percentage on the total values obtained (hereinafter reported as percentage of significative wavelengths, PSW).

**Figure 3:**
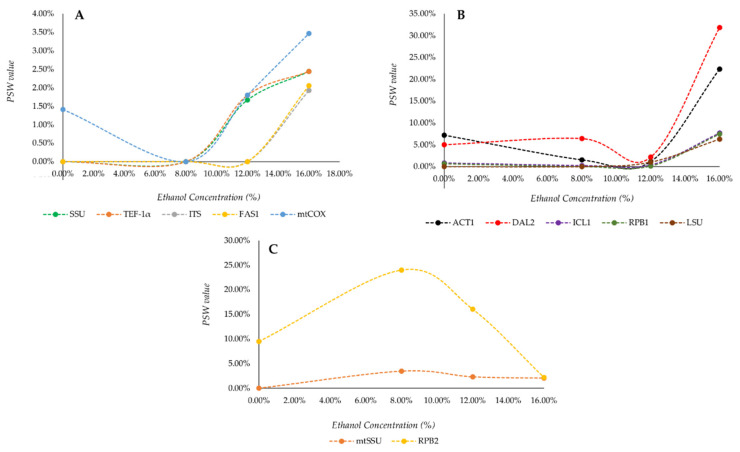
Trends of significative correlations between taxonomic markers and whole FTIR spectra from cells under short-term ethanol stress. Correlations between the distances of the IR spectra from cells challenged with four different ethanol concentrations (0%, 8%, 12% and 16%) and those obtained considering each taxonomic marker under study were reported as trends of Percentages of Significative Wavelengths (PSW). Markers were grouped according to their correlation trend in: low intensity responses (**A**); high intensity responses (**B**); and non-monotonous responses (**C**).

The low variability showed by LC-MS data did not make it possible to outline any defined relation with phylogenetic markers (data not shown). We therefore decided to concentrate the analysis on the only FTIR dataset by plotting the percentages of significative correlations with each marker in each tested condition, obtaining the trends reported in Figure 3.

Data presented in Figure 3 defined three different types of progression: (*i*) low intensity response (Figure 3A), characterized by monotonous trends and maximum PSW value between 2% and 4%; (*ii*) high intensity response (Figure 3B), characterized by monotonous trends and a maximum PSW value between 6% and 32%; and (*iii*) non-monotonous response (Figure 3C).

Low intensity responses were shown by SSU, *TEF-1α*, ITS, Beta subunit of fatty acid synthetase (*FAS1*) and mitochondrial COX (*mtCOXII*) genes (Figure 3A). The first four markers were characterized by an absence of correlations with 0% and 8% ethanol conditions; PSW values for SSU and *TEF-1α* showed then an increase to 1.67% and 1.8% at 12% ethanol and reached their maximum value (2.44%) in the last condition. A similar correlation rise was shown by ITS and *FAS1* markers after 12% ethanol exposure, reaching their maxima of 1.93% and 2.05%, respectively, at 16% ethanol.

The last marker with a low response is *mtCOXII*, characterized by a PSW value of 1.5% at 0% ethanol, followed by a decrease to zero and then a subsequent increase, similar to those of SSU and *TEF-1α*, to 1.80% at 12% ethanol. A maximum PSW value of 3.47% was finally reached in the last stressing condition.

High intensity response (Figure 3B) was shown by Actin encoding gene (*ACT1*), Allantoicase encoding gene (*DAL2*), *ICL1*, *RPB1* and LSU markers. These last three displayed very low PSW values at 0%, 8% and 12% ethanol stressing condition, reaching then their maxima of 7.70%, 7.45% and 6.29%, respectively, for 16% ethanol. *ACT1* and *DAL2* genes conversely showed, respectively, a PSW value of 7.19% and 5.01% in the resting condition and then slowly decreased, reaching zero at 12% ethanol; after that, PSW increased again, reaching values of 22.34% and 31.84% respectively.

The last type of response was that displayed by mitochondrial *SSU* and *RPB2*. Both trends showed a three-fold increase of PSW between 0% and 8% ethanol, followed by a gradual decrease to values of 2.05% and 2.18%, respectively, in correspondence of the last considered condition.

Noteworthy, ITS_LSU concatenation did not show in any case a correlation above the 0.75 threshold therefore its trend cannot be reported.

The analysis of these progressions has shown the existence of a certain correlation between some markers and the phenotypic response represented by the whole FTIR spectra. The subsequent step of the analysis focused then on the search for a more specific signal of the predictability of these markers towards the FTIR profiles.

**Figure 4:**
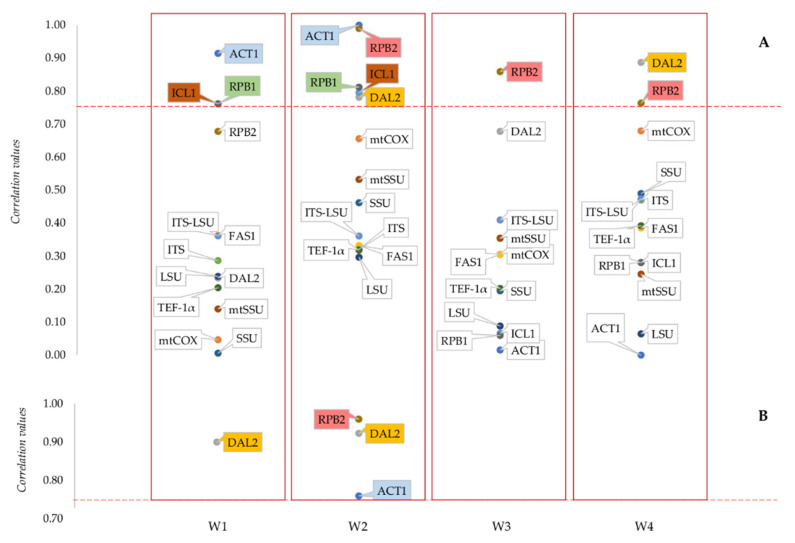
Correlations between taxonomic markers and characteristic spectral regions of the FTIR spectrum of both control and stressed cells. Maximum correlation values between each taxonomic marker and fatty acids (W1), amides (W2), mixed (W3) and carbohydrates (W4) spectral areas are reported for both control (**A**) and stressed (**B**) cells. The latter analysis was carried out grouping together data from the three stressing conditions tested. Colored labels refer to markers displaying correlation values higher than 0.75 threshold, represented by the red dashed lines.

Therefore, the correlation between the distances of the strains on the basis of the thirteen markers and the distances between the same strains calculated on the four specific spectral region related to stress response (Fatty acids W1, Amides W2, Mixed region W3 and Carbohydrates W4) considered separately was investigated. The spectra were divided into two groups: control cells spectra and ethanol stressed cells spectra, grouping together the data from the three stressing conditions (Figure 4 and Tables S11 and S12).

Maximum correlation values of each marker with the four IR regions are reported and quartiles thresholds are indicated as horizontal dashed lines. *ICL1*, *RPB1* and *ACT1* showed relatively high correlation values (0.76 for the first two and 0.91 for the third) with fatty acid (W1) region of control samples, followed by *RPB2*, placed just below 0.70. All the other markers showed lower correlation values, always below 0.40 (Figure 4A). The same four marker, together with *DAL2*, were highly correlated also with amides (W2) region of control samples.

Only *RPB2* and *DAL2* maintained a high correlation value with mixed (W3) and carbohydrates (W4) region, despite being the second marker slightly below 0.70 in W3 region; all the other markers showed correlation values around 0.5 or lower.

Noteworthy, the current official species markers ITS, LSU and their concatenation did not display significative correlations with the four IR regions, being always in the range between 0.25 and 0.5 for control samples.

Analyzing the correlation between the same markers and the 4 regions in the spectra of ethanol stressed cells (Figure 4B), a quite different picture is obtained. W1 region showed a correlation higher than 0.75 only with *DAL2* and *RPB2*, while the correlation with all the other markers was always lower.

The same two markers, together with *ACT1* and *mtCOXII*, were highly correlated also with W2 region. Interestingly, in this region, the correlation value for all the other markers was around 0.6, with the only exception of ITS_LSU concatenation, which showed a correlation around 0.4. The same decrease in correlation with W3 and W4 region observed for control cells spectra is present also in this second situation, in which all markers showed values below 0.75.

### 3.3. Phenotype Analysis in Response to Short-Term Ethanol Stress

**Figure 5:**
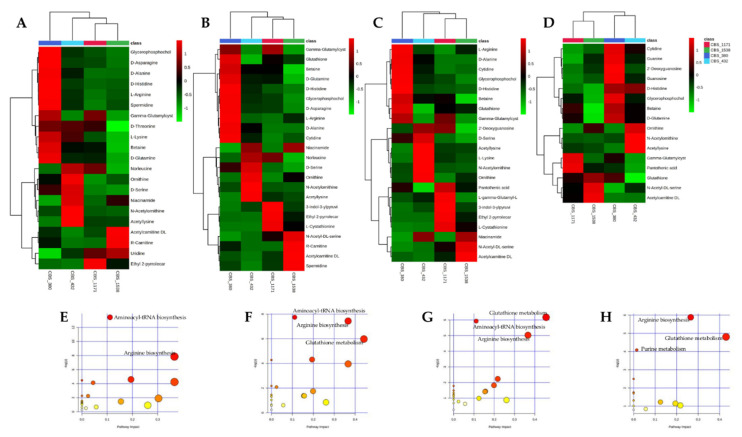
Heatmaps of the top significantly altered metabolites and pathways of *S. bayanus* CBS 380, *S. pastorianus* CBS 1538, *S. paradoxus* CBS 432 and *S. cerevisiae* 1171 strains after 1hr exposure to increasing ethanol concentrations. (**A**–**D**) Heatmaps of the top significantly altered metabolites at ethanol concentrations (*v*/*v*) of: 0% (**A**); 8% (**B**); 12% (**C**); and 16% (**D**). The colored boxes indicate the relative concentrations of the corresponding metabolite in each group under study. The color scale is log2 transformed value and indicates relatively high (red) and low (green) metabolite levels. (**E**–**H**) The most important altered pathways in the response of the four strains to ethanol (*v*/*v*) at: 0% (**E**); 8% (**F**); 12% (**G**); and 16% (**H**). y-axis, -log(p) represents metabolic pathways containing metabolites that are significantly different between the four strains; x-axis, pathway impact represents the impact of these significantly changed metabolites on the overall pathway, based on their position/number within the pathway.

LC-MS metabolomics was employed to assess the phenotypes of *S. bayanus*, *S. pastorianus*, *S. paradoxus* and *S. cerevisiae* type strains in response to short-term stress induced by ethanol. LC-MS was carried out on cell extracts from controls (0% ethanol) and treated cells at 8%, 12% and 16% ethanol (*v*/*v*), identifying a total of 89 metabolites with known structures (Table S1). To focus on the differentially altered metabolites between strains, Hierarchical Clustering Analysis (HCA) was performed according to the VIP scores from PLS-DA (Figure 5A–D).

Differentially altered metabolites were mainly amino acids and clustered separately into two main groups, defining a strain-specific pattern in each of the four conditions tested. Despite this evidence, the strains response at 8% and 12% ethanol (*v*/*v*) was very similar to that of controls, diverging significantly only at 16% ethanol. Interestingly, metabolites of the upper cluster were always upregulated in *S. bayanus* type strain, suggesting that the metabolome of this strain was significantly different from the others both in resting that in stressing conditions.

According to the results of significance of the perturbed metabolic pathway and the centrality of metabolites in the metabolic pathway, the perturbation of few metabolic pathways was found to be significant (Figure 5E–H).

Interestingly, two pathways distinguished the four strains regardless of the presence of ethanol. These differences were attributable to the upregulation of several metabolites in *S. bayanus* CBS 380 and *S. paradoxus* CBS 432, impacting mainly on the pathways of Aminoacyl-tRNA biosynthesis (d-Asparagine, d-Histidine, d-Arginine, d-Glutamine, d-Serine and d-Alanine) and Arginine biosynthesis (d-Arginine, d-Glutamine, *N*-Acetylornithine and d-Ornithine). Conversely, the only differences in cell metabolomes at increasing concentrations of ethanol concerned the differential regulation of metabolites such as Glutathione, gamma-l-Glutamyl-l-cysteine, l-Ornithine, Spermidine and (5-l-Glutamyl)-l-amino acid, all involved in the Gluthathione metabolism pathway.

## 4. Discussion

In the present study, we investigated the predictability of thirteen markers towards the complex FTIR and LC-MS metabolomic profiles of *S. cerevisiae*, *S. paradoxus*, *S. bayanus* and *S. pastorianus* reference strains. These *loci* were selected to check whether they could be used as predictors of specific physiological traits in addition to their taxonomic interest. The search of genes more correlated to ethanol stress, or other physiological features, is another type of work currently undergoing in our labs, in which all genes of the genomes are employed, independently of their taxonomic interest.

FTIR fingerprint has been proposed to classify and to identify species [32,33,43] and was able to detect sub-specific variations bound to the substrate of isolation (medical vs. food) more efficiently than DNA barcoding tools [44], whereas it was less efficient for the identification of strains of closely related species [45]. This evidence could explain the fact that no significant correlation was detected between molecular markers and FTIR profiles of strains under resting condition. Conversely, eight markers out of thirteen displayed correlation values over 0.75 with LC-MS data. DNA sequences accumulate mutations which then flow into proteome, whereas cell metabolism is the result of selective pressures occurred over a long time. The evolutionary changes in DNA, and consequently in proteins, describe relatively recent events compared to changes in metabolic pathways, which can be dated back in the past. It is therefore plausible that highly conserved sequences such as the mitochondrial genes *mtCOXII* and *mtSSU*; the traditional markers ITS and LSU and their concatenation ITS_LSU; and the coding sequences *TEF1α* and *FAS1* correlated highly with the LC-MS profiles of these four strains. These *loci* can therefore be proposed as “double usage” markers for taxonomy and general metabolic evolution.

The patterns of mortality under short-term ethanol stress confirmed what was previously described about the alcohol-tolerance of these species. Most strains of *S. cerevisiae*, *S. pastorianus* and *S. paradoxus* resulted tolerant up to 16% ethanol while *S. bayanus* strains showed serious difficulties withstanding 12% ethanol, and, when the percentage of ethanol increased up to 15%, the majority of strains were not able to grow [46]. The species of the *Saccharomyces sensu stricto* complex have all evolved into fermentation processes in highly challenging environments due to the presence of alcohol [47,48] supporting the hypothesis that functional associated traits have evolved slowly and are more phylogenetically conserved. In fact, most of the molecular markers showed increasing trends of correlation with FTIR profiles at increasing ethanol concentrations, confirming the primary role of the FTIR analytical system in finely characterizing the physiological status of cells in various conditions, including stress [18,49,50,51,52]. Several studies have shown that the transcription of *TEF-1α* can be induced by increasing stressing condition [53,54]. In addition, *FAS-1*, one of the main actors in yeast long-chain fatty acid metabolism, was interested in stress response to ethanol [55,56], antifoaming compounds [57] and high temperature [58], whereas the mitochondrial *COX* gene is a known to be part of the cellular response to different types of stressing agents, such as ethanol or high temperatures [59]. It has also been demonstrated that most of these genes play an important role in the response of yeasts to stressful fermentation conditions [56,60] and oxidative stress [61,62]. In light of these considerations, the coding sequences *mtCOXII*, *mtSSU*, *TEF-1α*, *FAS1*, *DAL2* and *ACT1* can be considered particularly interesting as putative markers for taxonomy and ethanol stress predictability. Further research in this area without restrictions regarding taxonomic markers is already underway to shed more light on this issue.

Finally, the results from pathways analysis suggest that the common ancestor of these species has evolved along the fermentation processes at high sugar concentrations and that the selective pressure that separated these species corresponded to a sugar concentration such as to produce around 12% (*v*/*v*) alcohol. These processes selected also the metabolisms of these organisms, leaving a specific trace in the two pathways of the Aminoacyl-tRNA and arginine biosynthesis. Many studies reported that, in *S. cerevisiae*, the amino acid arginine exerts a protective role against ethanol stress by maintaining the integrity of cell wall and plasma membrane [63] and by triggering the TCA cycle to provide more energy to contrast the stress [64]. On the other hand, the differential regulation of metabolites involved in the Glutathione metabolism looks like a strain-specific signal, linked to the participation of Glutathione of several cell functions, by searching for free radicals (ROS) present in the cytosol [65] and by playing a key role in inducible adaptive response mechanisms able to confer to cells the resistance to oxidative stress [65,66,67].

## 5. Conclusions

In this study, we aimed at establishing a new methodology in a well-known but restricted panel of strains and genes. The results presented prove the existence of a strong link between physiology and taxonomy suggesting that many *loci* could be particularly interesting as “double usage” markers for taxonomy and general metabolic evolution or ethanol stress. The approach is sound and can be replicated in other models, even with more components. On the other hand, we also showed that FTIR and LC-MS are complementary techniques that should be ideally deployed together. Reconnecting taxonomy with relevant phenotypic features represents an important challenge for microbiologists, the question on the real meaning of the species concept remaining open in light of the fact that most of the biotechnologically and industrially relevant characteristics of microbial cultures are encoded by quantitative trait loci (QTL). The application of artificial intelligence will surely improve the ability of omics tools in the species identification opening new scenarios to unravel this fascinating complexity.

## Figures and Tables

**Table 1:** Sequence IDs for the thirteen DNA markers used in the study.

Marker		Sequence ID			
Name	Acronym	CBS 380^T^ *S. bayanus*	CBS 432^T^ *S. paradoxus*	CBS 1171^T^ *S. cerevisiae*	CBS 1538^T^ *S. pastorianus*
Actin	*ACT1*	GCA_001515405.2	GCA_002079055.1	AF527913	GCA_001515465.2
Allantoicase	*DAL2*	GCA_001515405.2	GCA_002079055.1	S000001468	GCA_001515465.2
Beta subunit of fatty acid synthetase	*FAS1*	GCA_001515405.2	GCA_002079055.1	S000001665	GCA_001515465.2
Isocitrate lyase	*ICL1*	GCA_001515405.2	GCA_002079055.1	S000000867	GCA_001515465.2
Internal Transcribed Spacer	ITS	AY046152	AY046148	AY046146	AY046151
D1/D2 domain of rDNA Large Subunit (26S)	LSU D1/D2	U94931	U68555	U44806	AY048172
Mitochondrial subunit II of Cytochrome c oxidase	*mtCOXII*	GCA_001515405.2	GCA_002079055.1	GCA_000146045.2	GCA_001515465.2
Mitochondrial small ribosomal subunit	*mtSSU*	GCA_001515405.2	GCA_002079055.1	GCA_000146045.2	GCA_001515465.2
RNA polymerase II largest subunit	*RPB1*	GCA_001515405.2	GCA_002079055.1	AF527884	GCA_001515465.2
RNA polymerase II second largest subunit	*RPB2*	AY552472	AY552468	AY497600	GCA_001515465.2
rDNA Small Subunit (18S)	*SSU*	GCA_001515405.2	GCA_002079055.1	GCA_000146045.2	GCA_001515465.2
Translation Elongation Factor 1 alpha	*TEF1-α*	AF402012	AF402007	AF402004	AF402013
Concatenated ITS-LSU D1/D2	ITS-LSU	AY046152-U94931	AY046148-U68555	AY046146-U44806	AY046151-AY048172

**Table 2:** Mortality (%) induced by increasing ethanol concentrations on the four strains from *Saccharomyces sensu stricto* complex.

Ethanol% (*v*/*v*)	Mortality (%)			
	CBS 380^T^ *S. bayanus*	CBS 432^T^ *S. paradoxus*	CBS 1171^T^ *S. cerevisiae*	CBS 1538^T^ *S. pastorianus*
0	0	0	0	0
8	40.6	12.3	0.0	0.0
12	97.2	12.5	10.8	3.7
16	100.0	13.3	20.3	9.7

## Supplementary Materials

The following are available online at https://www.mdpi.com/2076-2607/8/8/1242/s1, Table S1: Metabolites identified for *S. bayanus* CBS 380, *S. pastorianus* CBS 1538, *S. paradoxus* CBS 432 and *S. cerevisiae* 1171 in response to short-term ethanol stress. Table S2: Important features identified by PLS-DA on control samples. VIP scores are calculated for each component. Table S3: Important features identified by PLS-DA on samples at 8% ethanol (*v*/*v*). VIP scores are calculated for each component. Table S4: Important features identified by PLS-DA on samples at 12% ethanol (*v*/*v*). VIP scores are calculated for each component. Table S5: Important features identified by PLS-DA on samples at 16% ethanol (*v*/*v*). VIP scores are calculated for each component. Table S6: Detailed results from pathway analysis. Comparison of the four strains at 0% ethanol (*v*/*v*). The statistical *p* values from enrichment analysis are further adjusted for multiple testing. Total is the total number of compounds in the pathway; Hits is the actually matched number from the user uploaded data; Raw *p* is the original *p* value calculated from the enrichment analysis; Holm *p* is the *p* value adjusted by Holm–Bonferroni method; FDR *p* is the *p* value adjusted using false discovery rate; Impact is the pathway impact value calculated from pathway topology analysis. Table S7: Detailed results from pathway analysis. Comparison of the four strains at 8% ethanol (*v*/*v*). The statistical *p* values from enrichment analysis are further adjusted for multiple testing. Total is the total number of compounds in the pathway; Hits is the actually matched number from the user uploaded data; Raw *p* is the original *p* value calculated from the enrichment analysis; Holm *p* is the *p* value adjusted by Holm–Bonferroni method; FDR *p* is the *p* value adjusted using false discovery rate; Impact is the pathway impact value calculated from pathway topology analysis. Table S8: Detailed results from pathway analysis. Comparison of the four strains at 12% ethanol (*v*/*v*). The statistical *p* values from enrichment analysis are further adjusted for multiple testing. Total is the total number of compounds in the pathway; Hits is the actually matched number from the user uploaded data; Raw *p* is the original *p* value calculated from the enrichment analysis; Holm *p* is the *p* value adjusted by Holm–Bonferroni method; FDR *p* is the *p* value adjusted using false discovery rate; Impact is the pathway impact value calculated from pathway topology analysis. Table S9: Detailed results from pathway analysis. Comparison of the four strains at 16% ethanol (*v*/*v*). The statistical *p* values from enrichment analysis are further adjusted for multiple testing. Total is the total number of compounds in the pathway; Hits is the actually matched number from the user uploaded data; Raw *p* is the original *p* value calculated from the enrichment analysis; Holm *p* is the *p* value adjusted by Holm–Bonferroni method; FDR *p* is the *p* value adjusted using false discovery rate Impact is the pathway impact value calculated from pathway topology analysis. Table S10: Percentage of significative wavelengths in the correlations between markers and IR bioassay conditions. Table S11: Correlation values of genetic markers and four IR regions in control samples; Table S12: Correlation values of genetic markers and four IR regions in ethanol stressed samples.

## References

[1] Guilliermond A. (1912). Les Levures.

[2] Kurtzman C.P., Robnett C.J. (1998). Identification and phylogeny of ascomycetous yeasts from analysis of nuclear large subunit (26S) ribosomal DNA partial sequences. Antonie Van Leeuwenhoek.

[3] Lodder J., Kreger-van Rij N.J.W. (1952). The yeasts—A taxonomic study. Mycology.

[4] Rosini G., Federici F., Vaughan A.E., Martini A. (1982). Systematics of the species of the yeast genus *Saccharomyces* associated with the fermentation industry. Appl. Microbiol. Biotechnol..

[5] Sipiczki M. (1989). Taxonomy and phylogenesis. Molecular Biology of the Fission Yeast.

[6] Blaxter M.L. (2004). The promise of a DNA taxonomy. Philos. Trans. R. Soc. B Biol. Sci..

[7] Schoch C.L., Seifert K.A., Huhndorf S., Robert V., Spouge J.L., Levesque C.A., Chen W., Bolchacova E., Voigt K., Crous P.W. (2012). Nuclear ribosomal internal transcribed spacer (ITS) region as a universal DNA barcode marker for Fungi. Proc. Natl. Acad. Sci. USA.

[8] Wijayawardene N.N., Hyde K.D., Rajeshkumar K.C., Hawksworth D.L., Madrid H., Kirk P.M., Braun U., Singh R.V., Crous P.W., Kukwa M. (2017). Notes for genera: Ascomycota. Fungal Divers..

[9] Li G.J., Hyde K.D., Zhao R.L., Hongsanan S., Abdel-Aziz F.A., Abdel-Wahab M.A., Alvarado P., Alves-Silva G., Ammirati J.F., Ariyawansa H.A. (2016). Fungal diversity notes 253–366: Taxonomic and phylogenetic contributions to fungal taxa. Fungal Divers..

[10] Ariyawansa H.A., Hyde K.D., Jayasiri S.C., Buyck B., Chethana K.T., Dai D.Q., Dai Y.C., Daranagama D.A., Jayawardena R.S., Lücking R. (2015). Fungal diversity notes 111-252-taxonomic and phylogenetic contributions to fungal taxa. Fungal Divers..

[11] Binati R.L., Junior W.J.L., Luzzini G., Slaghenaufi D., Ugliano M., Torriani S. (2020). Contribution of non-*Saccharomyces* yeasts to wine volatile and sensory diversity: A study on *Lachancea thermotolerans*, *Metschnikowia* spp. and *Starmerella bacillaris* strains isolated in Italy. Int. J. Food Microbiol..

[12] Ohta E., Nakayama Y., Mukai Y., Bamba T., Fukusaki E. (2016). Metabolomic approach for improving ethanol stress tolerance in *Saccharomyces cerevisiae*. J. Biosci. Bioeng..

[13] Paulo J.A., O’Connell J.D., Everley R.A., O’Brien J., Gygi M.A., Gygi S.P. (2016). Quantitative mass spectrometry-based multiplexing compares the abundance of 5000 *S. cerevisiae* proteins across 10 carbon sources. J. Proteom..

[14] Whitener M.E.B., Stanstrup J., Panzeri V., Carlin S., Divol B., Du Toit M., Vrhovsek U. (2016). Untangling the wine metabolome by combining untargeted SPME–GCxGC-TOF-MS and sensory analysis to profile Sauvignon blanc co-fermented with seven different yeasts. Metabolomics.

[15] Mendez K.M., Broadhurst D.I., Reinke S.N. (2019). The application of artificial neural networks in metabolomics: A historical perspective. Metabolomics.

[16] Damiani G., Grossi E., McCormick T., Cameron M., Cooper K. (2019). From Heat-Maps to Artificial Neural Networks: Multi-bioinformatics identify distinct subsets (endotypes) of psoriasis based on the metabolome of their uninvolved skin. J. Investig. Dermatol..

[17] Samaraweera M.A., Hall L.M., Hill D.W., Grant D.F. (2018). Evaluation of an artificial neural network retention index model for chemical structure identification in nontargeted metabolomics. Anal. Chem..

[18] Corte L., Rellini P., Roscini L., Fatichenti F., Cardinali G. (2010). Development of a novel, FTIR (Fourier Transform InfraRed spectroscopy) based, yeast bioassay for toxicity testing and stress response study. Anal. Chim. Acta.

[19] Kurtzman C., Fell J.W., Boekhout T. (2011). The Yeasts: A Taxonomic Study.

[20] Sharma R., Polkade A.V., Shouche Y.S. (2015). ‘Species concept’ in microbial taxonomy and systematics. Curr. Sci..

[21] Martini A.V. (1989). *Saccharomyces paradoxus* comb. nov., a Newly Separated Species of the *Saccharomyces sensu stricto* Complex Based upon nDNA/nDNA Homologies. Syst. Appl. Microbiol..

[22] Cardinali G., Martini A. (1994). Electrophoretic karyotypes of authentic strains of the *sensu stricto* group of the genus *Saccharomyces*. Int. J. Syst. Bacteriol..

[23] Goberna M., Verdu M. (2016). Predicting microbial traits with phylogenies. ISME J..

[24] Essendoubi M., Toubas D., Bouzaggou M., Pinon J.-M., Manfait M., Sockalingum G.D. (2005). Rapid identification of *Candida* species by FT-IR microspectroscopy. Biochim. Biophys. Acta Gen. Subj..

[25] Ramirez-Gaona M., Marcu A., Pon A., Guo A.C., Sajed T., Wishart N.A., Karu N., Feunang Y.D., Arndt D., Wishart D.S. (2016). YMDB 2.0: A significantly expanded version of the yeast metabolome database. Nucleic Acids Res..

[26] Kumar S., Stecher G., Li M., Knyaz C., Tamura K. (2018). MEGA X: Molecular evolutionary genetics analysis across computing platforms. Mol. Biol. Evol..

[27] Saitou N., Nei M. (1991). On the maximum-likelihood method for molecular phylogeny. J. Mol. Evol..

[28] Delcher A.L., Phillippy A., Carlton J., Salzberg S.L. (2002). Fast algorithms for large-scale genome alignment and comparison. Nucleic Acids Res..

[29] Delcher A.L., Kasif S., Fleischmann R.D., Peterson J., White O., Salzberg S.L. (1999). Alignment of whole genomes. Nucleic Acids Res..

[30] Li H., Handsaker B., Wysoker A., Fennell T., Ruan J., Homer N., Marth G., Abecasis G., Durbin R. (2009). The sequence alignment/map format and SAMtools. Bioinformatics.

[31] Antonielli L., Robert V., Corte L., Roscini L., Fatichenti A.B.F., Cardinali G. (2010). Searching for Related Descriptors Among Different Datasets: A New Strategy Implemented by the R Package’Dadi’. Open Appl. Inform. J..

[32] Helm D., Labischinski H., Schallehn G., Naumann D. (1991). Classification and identification of bacteria by Fourier-transform infrared spectroscopy. Microbiology.

[33] Naumann D., Helm D., Labischinski H. (1991). Microbiological characterizations by FT-IR spectroscopy. Nature.

[34] Chong J., Wishart D.S., Xia J. (2019). Using metaboanalyst 4.0 for comprehensive and integrative metabolomics data analysis. Curr. Protoc. Bioinform..

[35] Fernández E., Moreno F., Rodicio R. (1992). The *ICL1* gene from *Saccharomyces cerevisiae*. Eur. J. Biochem..

[36] Schöler A., Schüller H.J. (1994). A carbon source-responsive promoter element necessary for activation of the isocitrate lyase gene *ICL1* is common to genes of the gluconeogenic pathway in the yeast *Saccharomyces cerevisiae*. Mol. Cell. Biol..

[37] Schweizer M., Roberts L.M., Höltke H.-J., Takabayashi K., Höllerer E., Hoffmann B., Müller G., Köttig H., Schweizer E. (1986). The pentafunctional *FAS1* gene of yeast: Its nucleotide sequence and order of the catalytic domains. Mol. Genet. Genom..

[38] Yoo H.-S., Genbauffe F.S., Cooper T.G. (1985). Identification of the ureidoglycolate hydrolase gene in the *DAL* gene cluster of *Saccharomyces cerevisiae*. Mol. Cell. Biol..

[39] Kurtzman C.P. (2015). Identification of food and beverage spoilage yeasts from DNA sequence analyses. Int. J. Food Microbiol..

[40] Naseeb S., James S.A., Alsammar H., Michaels C.J., Gini B., Nueno-Palop C., Bond C.J., McGhie H., Roberts I.N., Delneri D. (2017). *Saccharomyces jurei* sp. nov., isolation and genetic identification of a novel yeast species from *Quercus robur*. Int. J. Syst. Evol. Microbiol..

[41] Weiss S., Samson F., Navarro D., Casaregola S. (2013). YeastIP: A database for identification and phylogeny of *Saccharomycotina* yeasts. FEMS Yeast Res..

[42] White T.J., Bruns T., Lee S., Taylor J. (1990). Amplification and direct sequencing of fungal ribosomal RNA genes for phylogenetics. PCR Protoc..

[43] Helm D., Naumann D. (1995). Identification of some bacterial cell components by FT-IR spectroscopy. FEMS Microbiol. Lett..

[44] Corte L., di Cagno R., Groenewald M., Roscini L., Colabella C., Gobbetti M., Cardinali G. (2015). Phenotypic and molecular diversity of *Meyerozyma guilliermondii* strains isolated from food and other environmental niches, hints for an incipient speciation. Food Microbiol..

[45] Colabella C., Corte L., Roscini L., Shapaval V., Kohler A., Tafintseva V., Tascini C., Cardinali G. (2017). Merging FT-IR and NGS for simultaneous phenotypic and genotypic identification of pathogenic *Candida* species. PLoS ONE.

[46] Belloch C., Orlic S., Barrio E., Querol A. (2008). Fermentative stress adaptation of hybrids within the *Saccharomyces sensu stricto* complex. Int. J. Food Microbiol..

[47] Borneman A.R., Pretorius I.S. (2015). Genomic insights into the *Saccharomyces sensu stricto* complex. Genetics.

[48] Sicard D., Legras J.-L. (2011). Bread, beer and wine: Yeast domestication in the *Saccharomyces sensu stricto* complex. Comptes Rendus Biol..

[49] Favaro L., Corte L., Roscini L., Cagnin L., Tiecco M., Colabella C., Berti A., Basaglia M., Cardinali G., Casella S. (2016). A novel FTIR-based approach to evaluate the interactions between lignocellulosic inhibitory compounds and their effect on yeast metabolism. RSC Adv..

[50] Mata-Miranda M.M., Guerrero-Ruiz M., Gonzalez-Fuentes J.R., Hernandez-Toscano C.M., Garcia-Andino J.R., Sanchez-Brito M., Vazquez-Zapien G.J. (2019). Characterization of the Biological Fingerprint and Identification of Associated Parameters in Stress Fractures by FTIR Spectroscopy. BioMed Res. Int..

[51] Da Cunha B.R., Fonseca L.P., Calado C.R.C. (2019). A phenotypic screening bioassay for *Escherichia coli* stress and antibiotic responses based on Fourier-transform infrared (FTIR) spectroscopy and multivariate analysis. J. Appl. Microbiol..

[52] Corte L., Roscini L., Pierantoni D.C., Pellegrino R.M., Emiliani C., Basaglia M., Favaro L., Casella S., Cardinali G. (2020). Delta-Integration of Single Gene Shapes the Whole Metabolomic Short-Term Response to Ethanol of Recombinant *Saccharomyces cerevisiae* Strains. Metabolites.

[53] Goud B.S., Ulaganathan K. (2019). RNA-seq analysis of transcriptomes for assessing stress tolerance of *S. cerevisiae* strain, NCIM3186. bioRxiv.

[54] Park H., Hwang Y.-S. (2008). Genome-wide transcriptional responses to sulfite in *Saccharomyces cerevisiae*. J. Microbiol..

[55] Diniz R.H.S., Villada J.C., Alvim M.C.T., Vidigal P.M.P., Vieira N.M., Lamas-Maceiras M., Cerdán M.E., González-Siso M.-I., Lahtvee P.-J., da Silveira W.B. (2017). Transcriptome analysis of the thermotolerant yeast *Kluyveromyces marxianus* CCT 7735 under ethanol stress. Appl. Microbiol. Biotechnol..

[56] Qiu Z., Jiang R. (2017). Improving *Saccharomyces cerevisiae* ethanol production and tolerance via RNA polymerase II subunit Rpb7. Biotechnol. Biofuels.

[57] Nielsen J.C., de Oliveira F.S.L., Rasmussen T.G., Thykær J., Workman C.T., Basso T.O. (2017). Industrial antifoam agents impair ethanol fermentation and induce stress responses in yeast cells. Appl. Microbiol. Biotechnol..

[58] Fu X., Li P., Zhang L., Li S. (2019). Understanding the stress responses of *Kluyveromyces marxianus* after an arrest during high-temperature ethanol fermentation based on integration of RNA-Seq and metabolite data. Appl. Microbiol. Biotechnol..

[59] Jiménez J., Benítez T. (1988). Yeast cell viability under conditions of high temperature and ethanol concentrations depends on the mitochondrial genome. Curr. Genet..

[60] Kaino T., Takagi H. (2008). Gene expression profiles and intracellular contents of stress protectants in *Saccharomyces cerevisiae* under ethanol and sorbitol stresses. Appl. Microbiol. Biotechnol..

[61] Du X., Takagi H. (2007). N-Acetyltransferase Mpr1 confers ethanol tolerance on *Saccharomyces cerevisiae* by reducing reactive oxygen species. Appl. Microbiol. Biotechnol..

[62] Dumond H., Danielou N., Pinto M., Bolotin-Fukuhara M. (2000). A large-scale study of Yap1p-dependent genes in normal aerobic and H2O2-stress conditions: The role of Yap1p in cell proliferation control in yeast. Mol. Microbiol..

[63] Cheng Y., Du Z., Zhu H., Guo X., He X. (2016). Protective effects of arginine on *Saccharomyces cerevisiae* against ethanol stress. Sci. Rep..

[64] Lourenco A.B., Roque F.C., Teixeira M.C., Ascenso J.R., Sá-Correia I. (2013). Quantitative 1H-NMR-metabolomics reveals extensive metabolic reprogramming and the effect of the aquaglyceroporin FPS1 in ethanol-stressed yeast cells. PLoS ONE.

[65] Grant C.M. (2001). Role of the glutathione/glutaredoxin and thioredoxin systems in yeast growth and response to stress conditions. Mol. Microbiol..

[66] Saharan R.K., Kanwal S., Sharma S.C. (2010). Role of glutathione in ethanol stress tolerance in yeast *Pachysolen tannophilus*. Biochem. Biophys. Res. Commun..

[67] Stephen D.W., Jamieson D.J. (1996). Glutathione is an important antioxidant molecule in the yeast *Saccharomyces cerevisiae*. FEMS Microbiol. Lett..

